# Clinical value of intratumoral and peritumoral CT radiomics models for discriminating benign and malignant parotid gland tumors

**DOI:** 10.3389/fonc.2025.1650943

**Published:** 2025-09-25

**Authors:** Cong Zhang, Naijing Shi, Yiru Wang, Mohan Hao, Jinwu Ren

**Affiliations:** ^1^ Department of Medical Imaging, The First Central Hospital of Baoding, Baoding, Hebei, China; ^2^ Graduate School, Chengde Medical University, Chengde, Hebei, China

**Keywords:** parotid gland tumor, radiomics, machine learning, intratumoral, peritumoral

## Abstract

**Objective:**

To evaluate the utility of combining unenhanced and contrast-enhanced CT intratumoral and peritumoral radiomic features with clinical variables for distinguishing benign from malignant parotid gland tumors.

**Methods:**

We retrospectively collected clinical and imaging data from 171 patients with pathologically confirmed parotid gland tumors treated at Baoding First Central Hospital between June 2019 and June 2025 (101 benign, 70 malignant). Tumor ROIs were manually delineated slice-by-slice on non-contrast, arterial-phase and venous-phase CT images, and peritumoral regions were automatically expanded by 1–4 mm. The cohort was randomly split into training and test sets at a 7:3 ratio. After extraction and selection of radiomic features, multiple models were constructed for intratumoral, various peritumoral ranges (1–4 mm) and intratumoral+peritumoral combinations. Model performance was evaluated by ROC curves, the optimal radiomics model was selected and integrated with the clinical model to produce a combined model, and a nomogram was subsequently developed.

**Results:**

The AUC values of the intratumoral, peritumoral (1–4 mm) and intratumoral+peritumoral models in the training set were 0.966, 0.953, 0.927, 0.983, 0.947, 0.959, 0.956, 0.909 and 0.976, respectively; in the test set the AUCs were 0.797, 0.766, 0.791, 0.714, 0.710, 0.805, 0.836, 0.778 and 0.753, respectively. According to the DeLong test, in the training set the differences between intratumor+peritumor 3mm *vs*. peritumor 3mm and between intratumor+peritumor 3mm *vs*. intratumor+peritumor 4mm were statistically significant (p = 0.022 and p = 0.026, respectively); in the test set, differences among the models were not statistically significant (P > 0.05). From this, it can be seen the combined intratumoral + 2 mm peritumoral radiomics model demonstrated superior diagnostic performance compared to models based exclusively on either intratumoral or peritumoral features. Consequently, this model was designated as the optimal radiomic signature and was integrated with independent clinical risk factors—specifically symptomatology and tumor margin status—to construct a combined clinical–radiomics predictive model. In the training and test sets, the AUC values of the radiomics model were 0.956 and 0.836, respectively, while those of the clinical model were 0.774 and 0.703. The combined model achieved AUC values of 0.974 and 0.844, demonstrating significantly superior diagnostic performance compared to the standalone clinical or radiomics models, along with the highest clinical utility. According to the Delong test, in the training set the differences between the clinical model and the combined model, and between the clinical model and the radiomics model, were statistically significant (p = 0.000 and p = 0.000, respectively); in the test set, differences among the models were not statistically significant (P > 0.05).

**Conclusion:**

A multiphase CT radiomics approach that fuses intratumoral features with a 2 mm peritumoral zone robustly distinguishes benign from malignant parotid gland tumors. Integration with key clinical predictors further enhances diagnostic accuracy, supporting clinical translation of the combined model for noninvasive tumor characterization.

## 1 Introduction

Salivary gland neoplasms represent one of the most common tumor entities within the head and neck region, of which parotid gland tumors (PGTs) account for approximately 80%, with nearly 20% exhibiting malignant behavior (1). Surgical excision remains the gold-standard treatment for PGTs; however, divergent histopathological subtypes necessitate tailored operative techniques and carry distinctly different prognoses (2). While benign parotid tumors generally confer favorable postoperative outcomes, malignant lesions demonstrate aggressive invasion, with a substantial risk of local recurrence and distant metastasis (3). Consequently, accurate preoperative discrimination between benign and malignant parotid lesions is of paramount clinical importance for guiding individualized treatment strategies.

Fine-needle aspiration biopsy (FNAB) is widely regarded as a reliable method for histological characterization of salivary gland masses (4). Nevertheless, FNAB is inherently invasive and may be complicated by hemorrhage, facial nerve injury, or acute inflammatory reactions (5, 6). Thus, noninvasive modalities capable of robustly distinguishing tumor malignancy are highly desirable. Conventional imaging techniques—including ultrasound, magnetic resonance imaging (MRI), and computed tomography (CT)—play pivotal roles in the diagnostic workup. Ultrasonography, as a first-line tool, effectively delineates cystic versus solid components and assesses lesion margins, yet its diagnostic yield is heavily operator-dependent and limited in evaluating deep lobe involvement. MRI offers superior soft-tissue contrast, and diffusion-weighted imaging (DWI) can aid in differentiating benign from malignant histology (7); however, its utility may be constrained by cost and contraindications such as metallic implants. CT reliably evaluates tumor margins, especially in the deep lobe, but demonstrates limited specificity for histopathological subtype discrimination (8). Although CT imaging involves ionizing radiation—especially salient in multiphasic scans and when contrast enhancement requires iodinated contrast agents—CT offers higher spatial resolution compared with MRI, which facilitates clearer delineation of tumor margins and thereby allows more precise definition of tumor extent and surrounding tissues.

In this context, there exists an urgent need for a more precise, noninvasive preoperative assessment method to differentiate benign from malignant parotid gland tumors. Radiomics—a quantitative imaging analysis technique that extracts high-dimensional features from medical images—has emerged as a promising approach to capture tumor microenvironment and heterogeneity, potentially reflecting underlying molecular and cellular characteristics (9). Recent studies have applied radiomics in head and neck malignancies (10), glioblastoma (11), breast cancer (12), and hepatocellular carcinoma (13), demonstrating strong associations between radiomic signatures and tumor histology, grade, and prognosis. Preliminary investigations into parotid tumors have leveraged radiomic features for lesion classification (14–16); however, most efforts have focused solely on intratumoral regions, overlooking the peritumoral microenvironment. Evidence suggests that peritumoral tissues may harbor crucial information regarding tumor invasiveness and heterogeneity (17). To date, few studies have systematically evaluated the peritumoral zone in radiomic analyses for parotid lesions, and optimal peritumoral margin size remains undetermined, often selected based on anecdotal experience or extrapolation from other tumor types.

Accordingly, the present study aims to develop and compare intratumoral and peritumoral CT radiomics models—across multiple peritumoral margins—derived from unenhanced and contrast-enhanced scans to distinguish benign from malignant parotid gland tumors. By integrating comprehensive radiomic profiling of both tumor and surrounding tissue, this work seeks to furnish clinicians with a more accurate and holistic noninvasive tool for preoperative decision-making.

## 2 Materials and methods

### 2.1 Patient cohort

**Figure 1:**
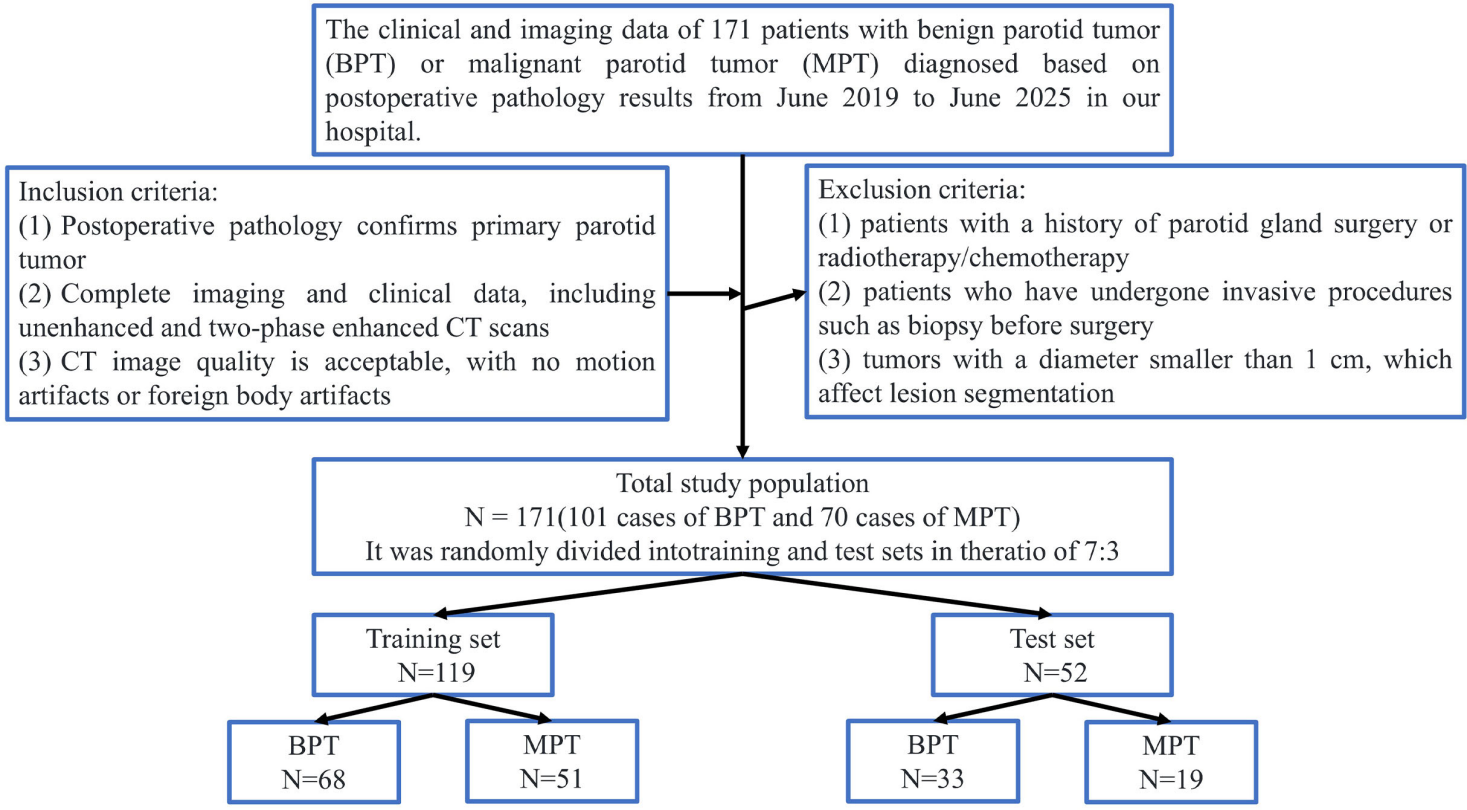
Flowchart of patient selection in this study.

Clinical and imaging data were retrospectively collected for 171 patients treated at The First Central Hospital of Baoding between June 2019 and June 2025 whose postoperative histopathology confirmed benign parotid gland tumors (BPT) or malignant parotid gland tumors (MPT). Inclusion criteria were: 1.histopathological diagnosis of primary parotid gland neoplasm; 2.availability of complete clinical records and high-quality CT scans, including unenhanced and two‐phase contrast-enhanced examinations; and 3.absence of significant motion or foreign‐body artifacts on CT images. Exclusion criteria comprised:1. prior parotid surgery or radiotherapy/chemotherapy; 2.history of invasive preoperative procedures such as biopsy; and 3. tumor diameter < 1 cm precluding reliable lesion segmentation (**Figure 1**). The cohort was randomly divided in a 7:3 ratio into a training set (n = 119; BPT = 68, MPT = 51) and a testing set (n = 52; BPT = 33, MPT = 19). The study protocol adhered to the Declaration of Helsinki and was approved by the Institutional Ethics Committee of The First Central Hospital of Baoding, with waiver of informed consent (Approval No. Kuai [2025]018).

### 2.2 Imaging acquisition

All examinations were performed using a Philips Brilliance i CT 128-slice scanner, covering bilateral parotid regions in the supine position. Scanning parameters were: 120 kV tube voltage, automatic tube current modulation, 1 mm slice thickness with 1 mm interslice interval, pitch 0.984:1, and gantry rotation time 0.6 s. Contrast enhancement was achieved via peripheral intravenous injection of iopamidol ( 300mg I/mL; 80–100 mL) at 3 mL/s using a dual-head power injector. Arterial-phase images were acquired 30–50 s after injection, and venous-phase images at 65–70 s. Patients were instructed to remove dentures and minimize swallowing during image acquisition.

### 2.3 Imaging and clinical data assessment

CT imaging features were independently evaluated in a blinded fashion by two radiologists (each with > 5 years of head and neck diagnostic experience) unaware of the clinical information and pathological results. In this study, two radiologists independently evaluated the imaging features of all 171 patients. The initial discordance rate between the two readers was approximately 12% (about 21 cases). All discrepancies were resolved successfully through consensus discussion between the two radiologists, and a final unanimous assessment was reached. Imaging features included tumor location (left/right), margin definition (well-defined/ill-defined), shape (regular/irregular), deep-lobe involvement (determined by the imaginary line connecting the most dorsal point of the posterior facial vein to the most dorsal point of the ipsilateral mastoid process), calcification (present/absent), attenuation homogeneity (homogeneous/heterogeneous), lymph node enlargement (present/absent), maximum tumor diameter, peak enhancement phase (arterial/venous), and enhancement uniformity (homogeneous/heterogeneous). Clinical variables comprised sex, age, smoking history, alcohol consumption, and presence of clinical symptoms.

### 2.4 Image segmentation

**Figure 2:**
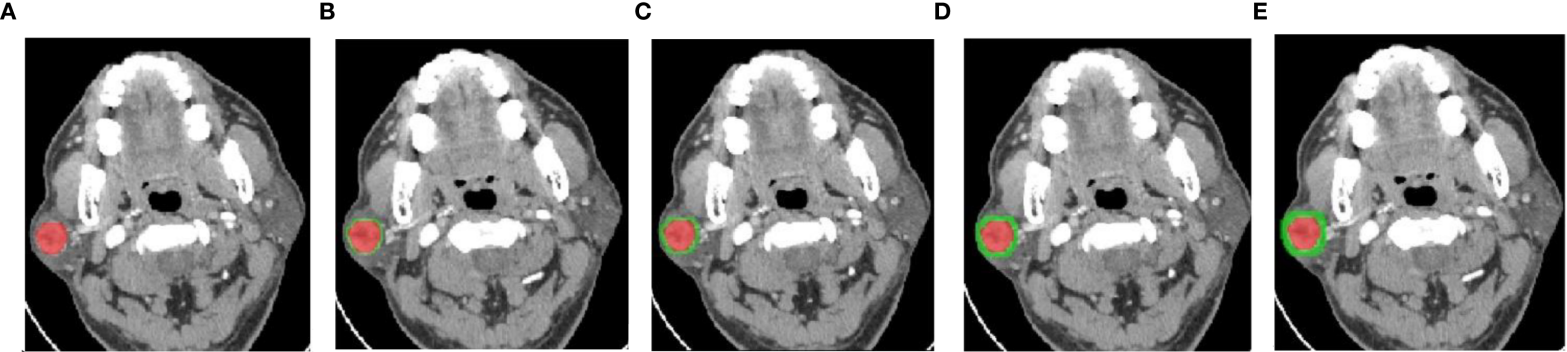
Intratumoral ROI **(A)**, intratumoral plus peritumoral 1mm ROI **(B)**, intratumoral plus peritumoral 2mm ROI **(C)**, intratumoral plus peritumoral 3mm ROI **(D)**, and intratumoral plus peritumoral 4mm ROI **(E)**.

Three‐phase CT images were exported from the PACS system and resampled to isotropic voxels of 1 mm × 1 mm × 1 mm; all image intensities were then normalized. A radiologist blinded to the pathological results manually delineated the tumor region of interest (ROI) on axial unenhanced, arterial‐phase, and venous‐phase images using ITK‐SNAP software, carefully excluding bone and vessels. The intratumoral ROI was subsequently expanded outward by 1 mm, 2 mm, 3 mm, and 4 mm to generate peritumoral ROIs (workflow shown in **Figure 2**); these were labeled Intra, Peri1mm, Peri2mm, Peri3mm, and Peri4mm. ROI consistency was maintained by first outlining the arterial‐phase tumor ROI, then applying the same boundaries to the unenhanced and venous‐phase ROIs, followed by manual adjustment to ensure accuracy. To assess segmentation reproducibility, a subset of 30 patients was randomly selected one month later for repeat delineation, and the intraclass correlation coefficient (ICC) was calculated.

### 2.5 Feature extraction and selection

Radiomic features were extracted using PyRadiomics v3.0.1. Features with ICC ≥ 0.75 were retained and standardized by z-score normalization. Univariate analysis (Student’s t-test or Mann–Whitney U test) and Pearson correlation filtering were performed, followed by least absolute shrinkage and selection operator (LASSO) logistic regression with ten-fold cross-validation to optimize the penalty parameter and select the most robust predictors.

### 2.6 Model construction

Clinical–imaging predictors significantly associated with BPT and MPT were identified by univariate and multivariate logistic regression to build the clinical model. Given prior evidence that CT-based machine learning using a support vector machine (SVM) classifier achieves high accuracy for parotid tumor discrimination (18), SVM algorithms were employed to develop nine radiomics models: Intra; Peri1mm; Peri2mm; Peri3mm; Peri4mm; Intra + Peri1mm; Intra + Peri2mm; Intra + Peri3mm; and Intra + Peri4mm. Receiver operating characteristic (ROC) curves were plotted and area under the curve (AUC) values calculated to evaluate each model’s performance in differentiating benign from malignant parotid lesions. The optimal radiomics model was then combined with the clinical model to construct a joint model, which was visualized as a nomogram.

### 2.7 Statistical analysis

**Figure 3:**
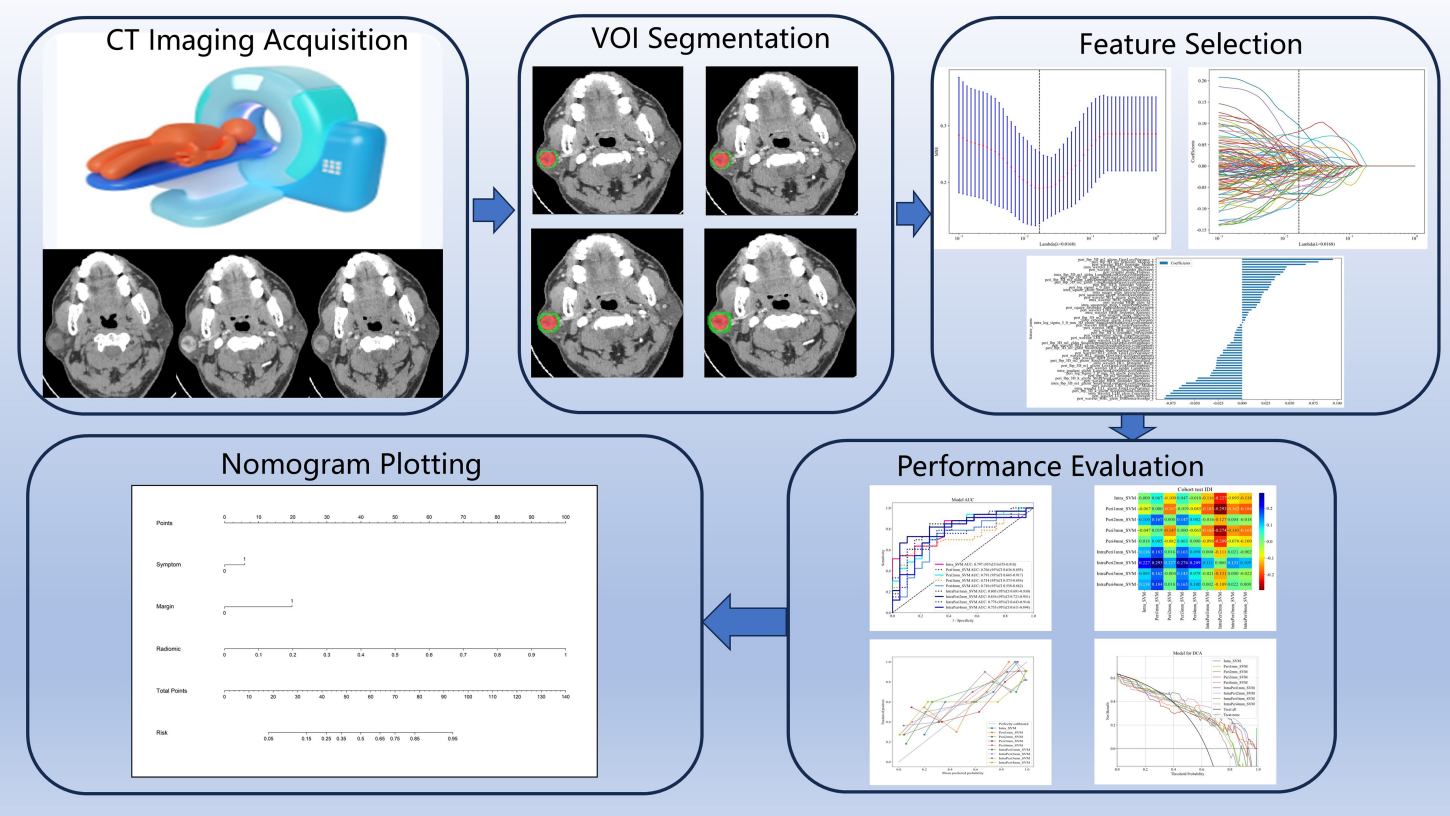
Radiomics analysis workflow of this study.

Clinical and imaging characteristics of the training and validation cohorts were analyzed using Python 3.7.0 with statsmodels v0.13. Continuous variables were compared by independent‐samples t-test or Mann–Whitney U test, and categorical variables by χ² test; P < 0.05 was considered statistically significant. Diagnostic performance of each model was assessed by ROC analysis, reporting AUC, accuracy, sensitivity, specificity, positive predictive value (PPV), and negative predictive value (NPV). Delong’s test was used to compare AUCs between models. Calibration performance was evaluated via calibration curves, and clinical utility was appraised using decision curve analysis (DCA). The overall radiomics workflow is depicted in **Figure 3**.

## 3 Results

### 3.1 Clinical data and imaging features

This study enrolled 171 patients, of whom 101 were pathologically confirmed as having benign parotid tumors (BPT) and 70 as malignant parotid tumors (MPT); the cohort was randomly split 7:3 into a training set of 119 patients (68 BPT, 51 MPT) and a test set of 52 patients (33 BPT, 19 MPT). The detailed clinical and imaging characteristics are summarized in **Table 1**. There were no statistically significant differences between BPT and MPT groups in age, sex, smoking history, alcohol history, maximum tumor diameter, shape, location, distribution, calcification, attenuation homogeneity, peak enhancement phase, or enhancement uniformity (all P > 0.05). Furthermore, univariate and multivariate logistic regression analyses identified clinical symptoms and tumor margin status as independent predictors of malignancy (P < 0.05; see **Table 2**), upon which the clinical model was built, yielding AUCs of 0.774 and 0.703 in the training and test sets, respectively.

**Table 1:** Baseline characteristics of patients in training and validation set.

Variable	Train set (n=119)		pvalue	Test set (n=52)		pvalue
	MPT (n=51)	BPT (n=68)		MPT (n=19)	BPT (n=33)	
Age	56.35 ± 17.09	55.41 ± 14.44	0.586	57.05 ± 14.83	56.24 ± 14.91	0.79
_Maximum_diameter	3.19 ± 1.52	2.89 ± 1.19	0.367	3.21 ± 1.78	2.61 ± 1.04	0.387
Gender			0.935			1
0	19 (37.25)	27 (39.71)		8 (42.11)	13 (39.39)	
1	32 (62.75)	41 (60.29)		11 (57.89)	20 (60.61)	
Smoking			0.145			1
0	31 (60.78)	31 (45.59)		10 (52.63)	17 (51.52)	
1	20 (39.22)	37 (54.41)		9 (47.37)	16 (48.48)	
_Drinking			0.246			1
0	34 (66.67)	37 (54.41)		10 (52.63)	17 (51.52)	
1	17 (33.33)	31 (45.59)		9 (47.37)	16 (48.48)	
Symptom			<0.001			<0.001
0	19 (37.25)	52 (76.47)		9 (47.37)	31 (93.94)	
1	32 (62.75)	16 (23.53)		10 (52.63)	2 (6.06)	
Location			0.615			0.249
0	23 (45.10)	35 (51.47)		7 (36.84)	19 (57.58)	
1	28 (54.90)	33 (48.53)		12 (63.16)	14 (42.42)	
Margin			<0.001			0.148
0	31 (60.78)	14 (20.59)		7 (36.84)	5 (15.15)	
1	20 (39.22)	54 (79.41)		12 (63.16)	28 (84.85)	
Shape			0.038			1
0	42 (82.35)	43 (63.24)		12 (63.16)	20 (60.61)	
1	9 (17.65)	25 (36.76)		7 (36.84)	13 (39.39)	
Involving_deep_leaves			0.811			1
0	22 (43.14)	32 (47.06)		11 (57.89)	20 (60.61)	
1	29 (56.86)	36 (52.94)		8 (42.11)	13 (39.39)	
Calcification			0.21			1
0	47 (92.16)	67 (98.53)		18 (94.74)	31 (93.94)	
1	4 (7.84)	1 (1.47)		1 (5.26)	2 (6.06)	
Density			0.109			1
0	36 (70.59)	37 (54.41)		12 (63.16)	21 (63.64)	
1	15 (29.41)	31 (45.59)		7 (36.84)	12 (36.36)	
Swollen_lymph_nodes			<0.001			0.018
0	14 (27.45)	46 (67.65)		6 (31.58)	23 (69.70)	
1	37 (72.55)	22 (32.35)		13 (68.42)	10 (30.30)	
Enhanced_peak_phase			1			0.739
0	32 (62.75)	43 (63.24)		11 (57.89)	22 (66.67)	
1	19 (37.25)	25 (36.76)		8 (42.11)	11 (33.33)	
_Enhanced_uniformity			0.013			0.002
0	27 (52.94)	52 (76.47)		10 (52.63)	31 (93.94)	
1	24 (47.06)	16 (23.53)		9 (47.37)	2 (6.06)	

**Table 2:** Univariable and multivariable logistic regression analysis of factors.

Variable	Univariate analysis		Multivariate analysis	
	OR (95% CI)	p_value	OR (95% CI)	p_value
Calcification	0.250 (0.040-1.573)	0.215		
Symptom	0.500 (0.302-0.827)	0.024	0.155 (0.077-0.313)	0
Swollen_lymph_nodes	0.595 (0.382-0.926)	0.053		
Enhanced_uniformity	0.667 (0.392-1.133)	0.209		
Age	1.005 (0.999-1.010)	0.159		
Maximum_diameter	1.053 (0.961-1.154)	0.356		
Location	1.179 (0.773-1.799)	0.523		
Involving_deep_leaves	1.241 (0.824-1.872)	0.386		
Gender	1.281 (0.869-1.889)	0.293		
Enhanced_peak_phase	1.316 (0.798-2.171)	0.367		
Drinking	1.824 (1.110-2.995)	0.047	0.851 (0.308-2.356)	0.795
Smoking	1.850 (1.172-2.921)	0.027	2.188 (0.792-6.044)	0.205
Density	2.067 (1.232-3.466)	0.021	0.963 (0.428-2.166)	0.939
Margin	2.700 (1.756-4.154)	0	3.891 (1.831-8.265)	0.003
Shape	2.778 (1.465-5.265)	0.009	1.236 (0.495-3.083)	0.704

### 3.2 Radiomics model development

**Figure 4:**
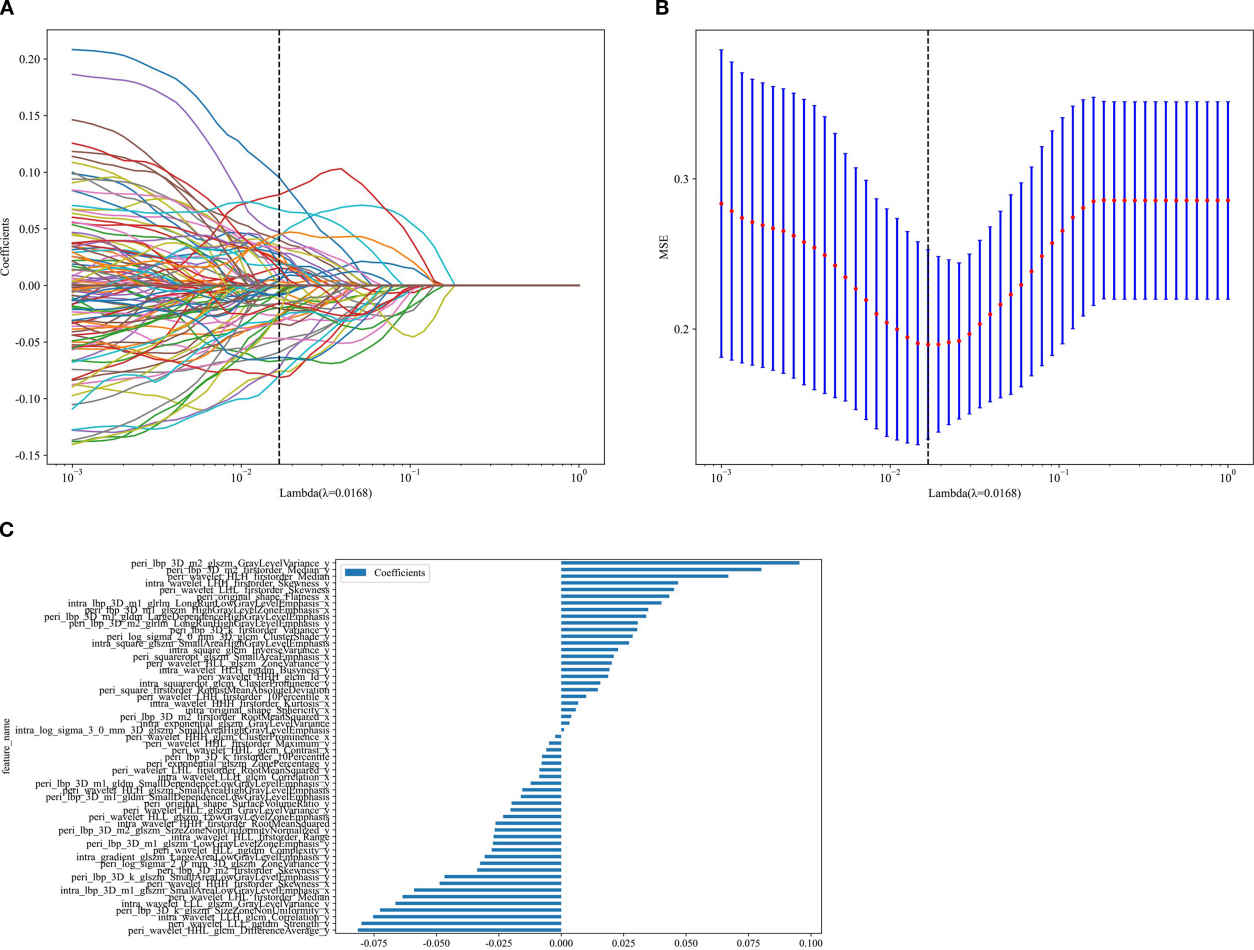
Process of extracting radiomics features. **(A)** The vertical axis of LASSO regression represents the coefficients corresponding to 5502 features, and the horizontal axis represents the adjustment parameter λ; **(B)** The optimal λ (λ=0.0168) is selected through mRMR; **(C)** 56 IntraPeri optimal features have been extracted. IntraPeri, Intratumoral + 2mm peritumoral combined model; LASSO, least absolute shrinkage and selection operator; mRMR, maximum relevance-minimum redundancy.

**Figure 5:**
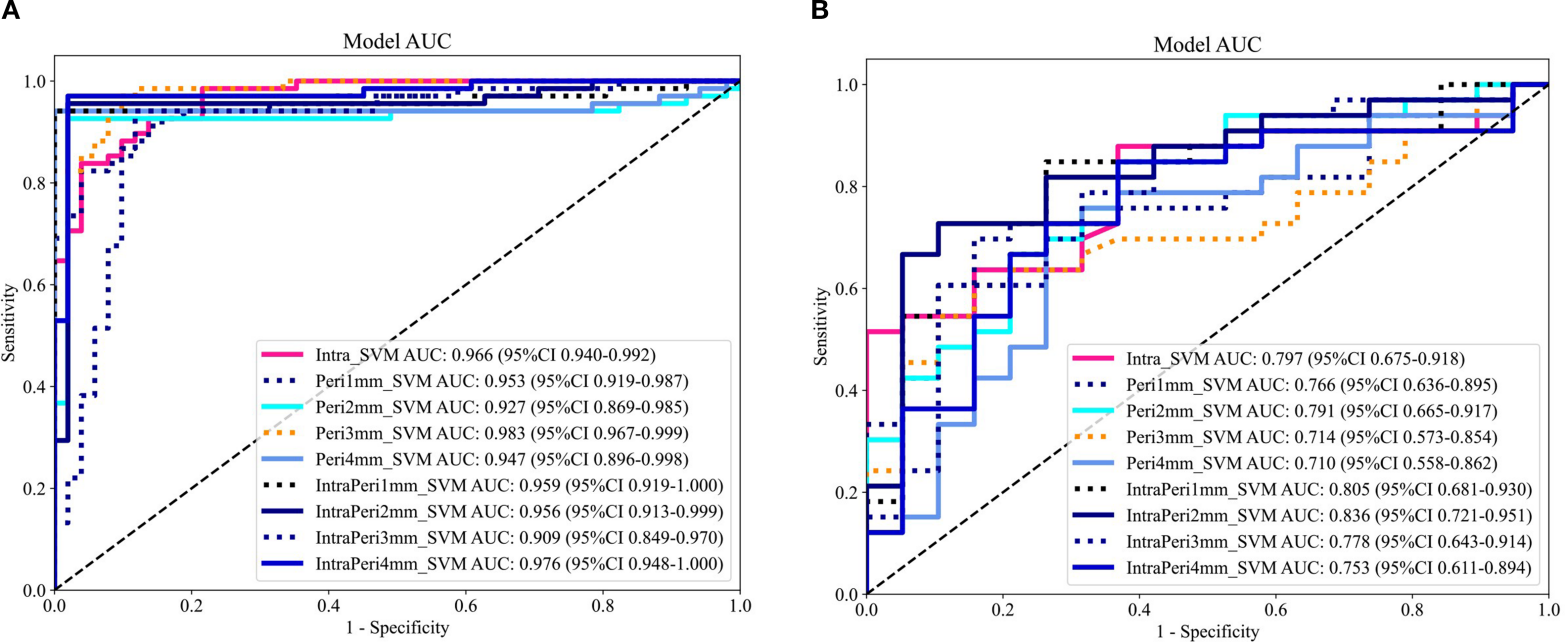
ROC curves of nine radiomic models **(A)** training set; **(B)** test set.

**Figure 6:**
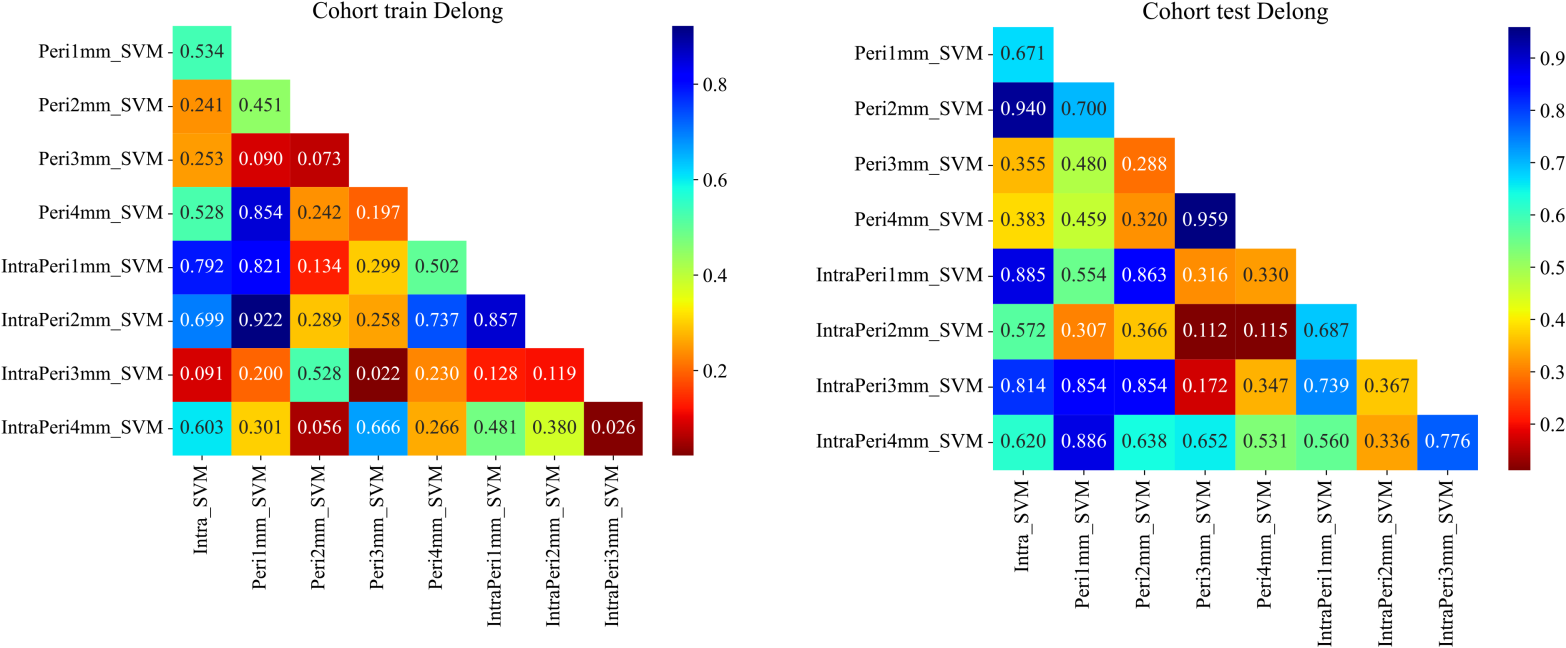
Summary of DeLong test results for each model.

A total of 1,834 radiomic features per ROI were extracted using PyRadiomics v3.0.1, comprising 360 first-order features, 14 shape features, and 1,460 texture features (440 gray-level co-occurrence matrix, 280 gray-level dependence matrix, 320 gray-level run-length matrix, 320 gray-level size-zone matrix, and 14 neighborhood gray-tone difference matrix features), resulting in 5,502 features across the three CT phases. After LASSO selection, 20, 33, 39, 34, and 60 features were retained for the Intra, Peri1 mm, Peri2 mm, Peri3 mm, and Peri4 mm ROIs respectively (see **Figure 4**). In both the training and test cohorts, the combined intratumoral + 2 mm peritumoral model achieved AUCs of 0.956 and 0.836, outperforming all other radiomics models (**Table 3**). The ROC curves for all nine radiomics models are displayed in **Figure 5**; Thus, the intratumoral + 2 mm peritumoral model emerged as the optimal radiomics signature, According to the Delong test, in the training set the differences between intratumor+peritumor 3mm *vs*. peritumor 3mm and between intratumor+peritumor 3mm *vs*. intratumor+peritumor 4mm were statistically significant (p = 0.022 and p = 0.026, respectively); in the test set, differences among the models were not statistically significant (P > 0.05). **Figure 6** illustrates the Delong test results for each model.

**Table 3:** The predictive performance of the nine models.

Models	Cohort	AUC	95% CI	Accuracy	Sensitivity	Specificity	PPV	NPV
Intra_SVM	train	0.966	0.9398 - 0.9922	0.891	0.838	0.961	0.966	0.817
	test	0.797	0.6754 - 0.9179	0.692	0.515	0.895	0.895	0.543
Peri1mm_SVM	train	0.953	0.9195 - 0.9868	0.882	0.824	0.961	0.966	0.803
	test	0.766	0.6357 - 0.8954	0.750	0.697	0.842	0.885	0.615
Peri2mm_SVM	train	0.927	0.8692 - 0.9849	0.950	0.926	0.98	0.984	0.909
	test	0.791	0.6651 - 0.9170	0.769	0.848	0.632	0.800	0.706
Peri3mm_SVM	train	0.983	0.9666 - 0.9994	0.941	0.971	0.902	0.930	0.958
	test	0.714	0.5734 - 0.8540	0.692	0.606	0.842	0.870	0.552
Peri4mm_SVM	train	0.947	0.8964 - 0.9981	0.966	0.941	0.929	0.92	0.927
	test	0.710	0.5579 - 0.8616	0.731	0.727	0.737	0.828	0.609
IntraPeri1mm_SVM	train	0.959	0.9186 - 1.0000	0.966	0.941	0.892	0.918	0.927
	test	0.805	0.6806 - 0.9303	0.808	0.848	0.737	0.848	0.737
IntraPeri2mm_SVM	train	0.956	0.9126 - 0.9992	0.966	0.956	0.980	0.985	0.943
	test	0.836	0.7209 - 0.9505	0.788	0.727	0.895	0.923	0.654
IntraPeri3mm_SVM	train	0.909	0.8486 - 0.9698	0.916	0.941	0.882	0.914	0.918
	test	0.778	0.6431 - 0.9136	0.712	0.606	0.895	0.909	0.567
IntraPeri4mm_SVM	train	0.976	0.9479 - 1.0000	0.975	0.971	0.980	0.985	0.962
	test	0.753	0.6114 - 0.8942	0.769	0.848	0.632	0.800	0.706

### 3.3 Nomogram construction

**Figure 7:**
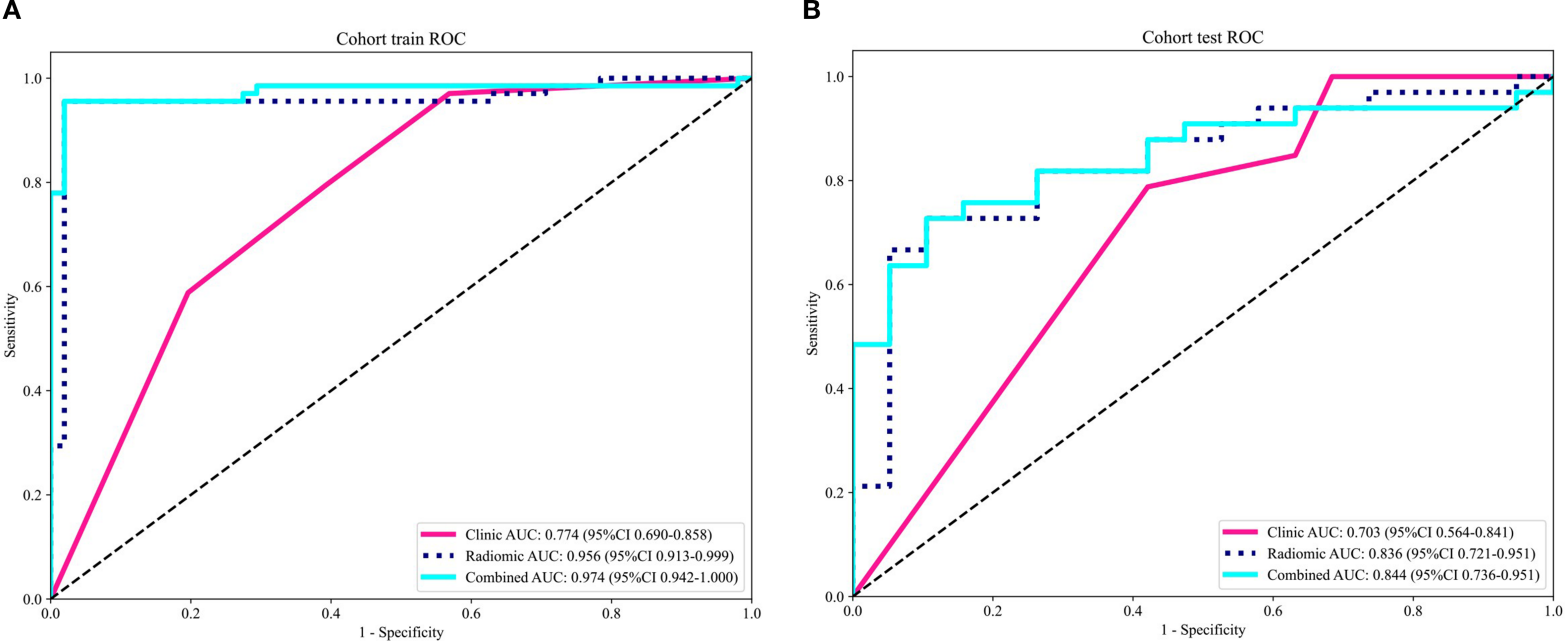
ROC curves demonstrating the discriminatory performance of radiomics, clinical, and integrated models in parotid gland tumor classification **(A)** training set; **(B)** test set.

**Figure 8:**
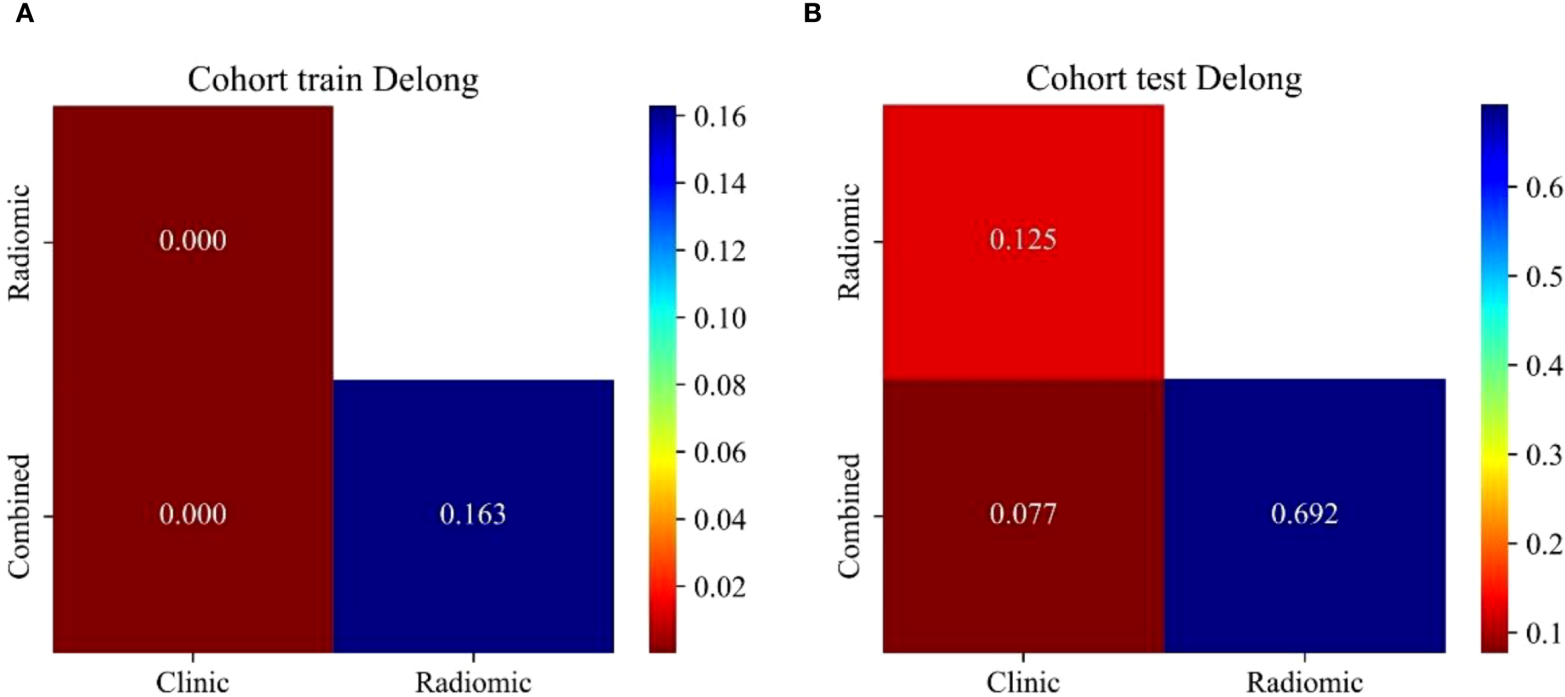
Delong test results comparing the performance of clinical, radiomics, and combined models.

**Figure 9:**
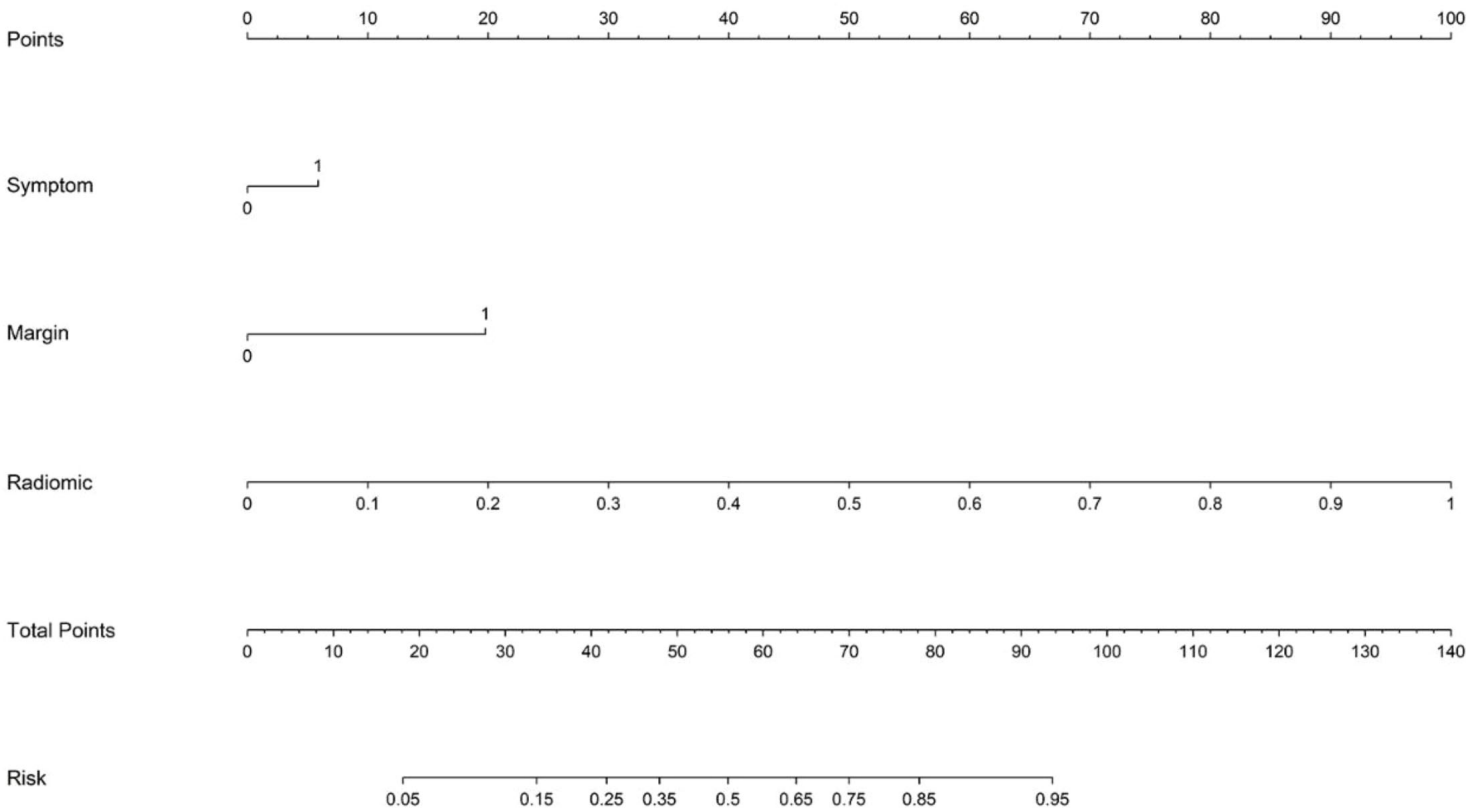
Nomogram of the combined model.

**Figure 10:**
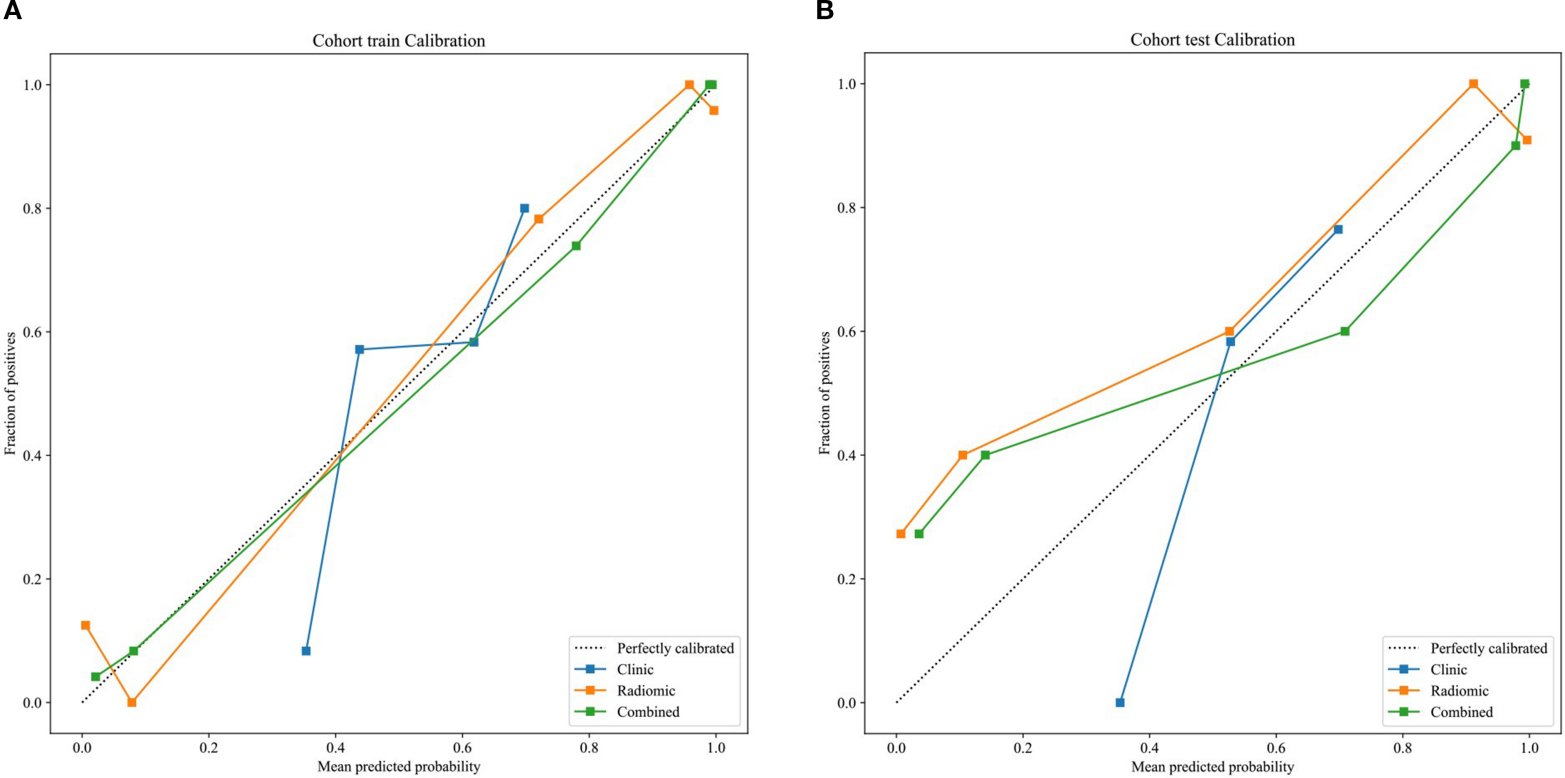
calibration curves of clinical, radiomics, and nomogram models **(A)** training set; **(B)** test set.

**Figure 11:**
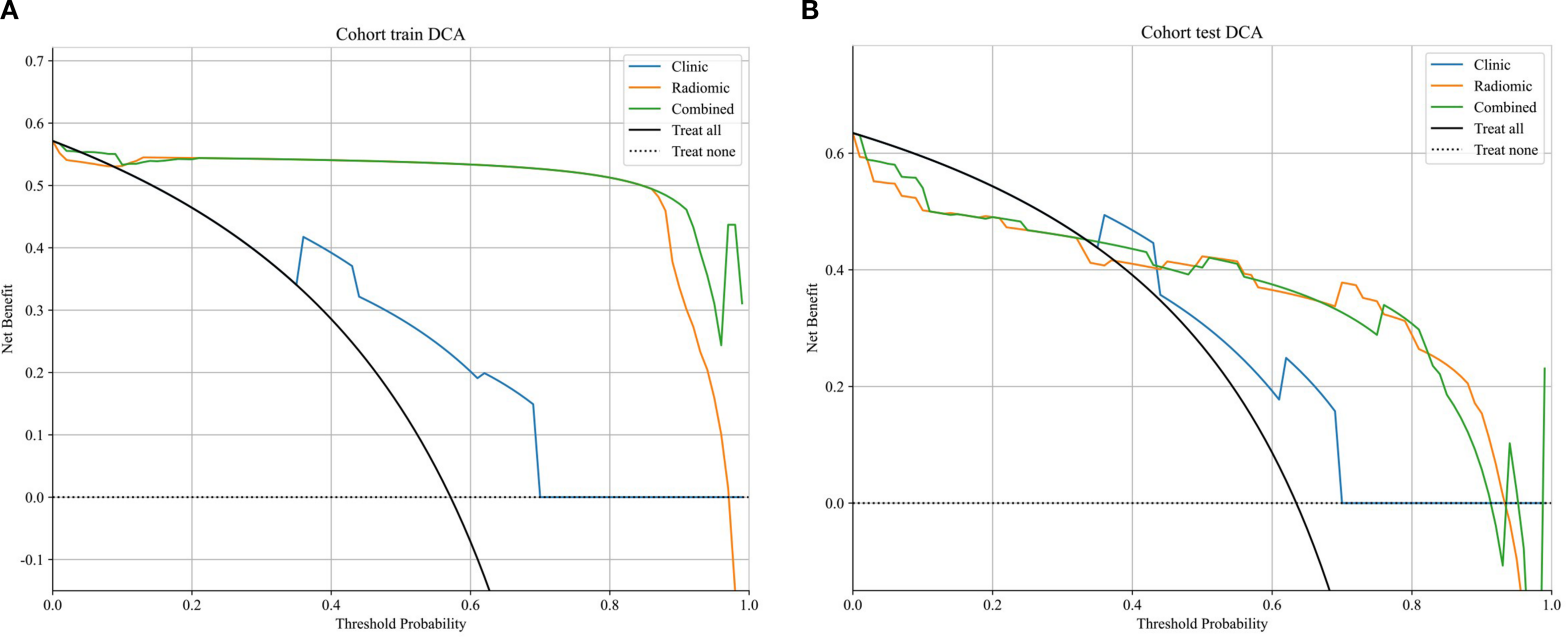
DCA curves of clinical, radiomics, and nomogram models **(A)** training set; **(B)** test set.

The optimal radiomics model (Intra + Peri2 mm) was combined with the clinical model to generate a nomogram (joint model) as detailed in **Table 4** and **Figure 7**. In the training and test sets, the radiomics model AUCs were 0.956 and 0.836, respectively, while the clinical model yielded AUCs of 0.774 and 0.703. The combined model demonstrated AUC values of 0.974 and 0.844, surpassing those of the individual radiomics and clinical models, and indicating superior diagnostic performance. According to the Delong test, in the training set the differences between the clinical model and the combined model, and between the clinical model and the radiomics model, were statistically significant (p = 0.000 and p = 0.000, respectively); in the test set, differences among the models were not statistically significant (P > 0.05). Delong’s comparisons among the three models are shown in **Figure 8**. The nomogram assigns substantial weight to the radiomics score in malignancy risk estimation (**Figure 9**). Calibration curves for all three models in both cohorts confirmed excellent goodness-of-fit (**Figure 10**), with Hosmer–Lemeshow P values of 0.392 and 0.435 for the training and test sets, respectively, indicating no significant deviation from perfect calibration. Decision curve analysis (**Figure 11**) demonstrated that the joint model achieved the highest net clinical benefit for parotid tumor discrimination in both cohorts.

**Table 4:** Diagnostic efficacy of clinical, radiomics, and nomogram models in both training and testing cohorts.

Model	Cohort	AUC	95% CI	Accuracy	Sensitivity	Specificity	PPV	NPV
Clinic	train	0.774	0.6903 - 0.8576	0.739	0.971	0.431	0.695	0.917
Clinic	test	0.703	0.5642 - 0.8409	0.712	0.788	0.579	0.765	0.611
Radiomic	train	0.956	0.9126 - 0.9992	0.966	0.956	0.98	0.985	0.943
Radiomic	test	0.836	0.7209 - 0.9505	0.788	0.727	0.895	0.923	0.654
Combined	train	0.974	0.9424 - 1.0000	0.966	0.956	0.98	0.985	0.943
Combined	test	0.844	0.7364 - 0.9510	0.788	0.727	0.895	0.923	0.654

## 4 Discussion

Benign and malignant parotid gland tumors exhibit markedly distinct biological behaviors, leading to divergent treatment strategies and prognosis. For benign lesions, superficial or partial parotidectomy is typically preferred; by contrast, malignant tumors are associated with higher rates of recurrence and metastasis and often warrant more extensive resection, such as total or radical parotidectomy (19). Consequently, accurate preoperative differentiation between benign and malignant tumors is critical for optimizing therapeutic decision-making and patient outcomes. Conventional imaging assessments rely heavily on the radiologist’s expertise and subjective interpretation, and overlapping radiographic features among different histological subtypes further limit diagnostic precision (20). As a result, the accuracy of preoperative characterization of parotid tumors remains suboptimal (21).

Radiomics—an emerging artificial intelligence–driven methodology—enables noninvasive extraction of high-dimensional quantitative features from routine medical images, thereby revealing intrinsic tumor biology (22). Prior studies have predominantly focused on intratumoral radiomic signatures (23, 24), with comparatively little attention paid to the peritumoral microenvironment. Xu et al. demonstrated the utility of tumor-based radiomic features for distinguishing benign from malignant parotid lesions (25). Zheng et al. constructed an MRI-based radiomics nomogram for preoperative differentiation of benign and malignant parotid tumors. By extracting texture features from T1WI and fat-suppressed T2WI and integrating clinical factors (such as deep-lobe invasion and peritumoral tissue infiltration), the model demonstrated high discriminative performance in both training and validation cohorts (AUC > 0.93). The pathological basis is that malignant tumors often demonstrate infiltrative growth and high tissue heterogeneity—attributes that can be quantitatively captured by radiomic features—thus surpassing visual assessment and aiding clinical decision-making (26). Faggioni et al. demonstrated that MRI-based radiomic features—particularly T2-weighted Skewness and pcsT1-weighted GLCM_InverseVariance—can effectively discriminate pleomorphic adenoma (PA) from Warthin tumor (WT), exhibiting high specificity. One study found that on contrast-enhanced T1-weighted images, GLCM_InverseVariance was the most discriminative feature (AUC=0.90), reflecting the relatively homogeneous enhancement pattern of WT; on T2-weighted images, skewness had the highest specificity (88%), with high skewness values associated with intralesional cystic change, necrosis, or hypervascularity typical of WT. WT commonly contains cystic components and lymphoid stroma with complex architecture, whereas PA is relatively more homogeneous; these histopathologic differences are quantitatively captured by radiomic features, enabling noninvasive and accurate preoperative discrimination (27). Evidence suggests that the peritumoral zone harbors critical biological information—such as angiogenesis, lymphovascular invasion, and stromal reactions—that may drive tumor progression (28, 29). These pathophysiological changes can likewise be captured through radiomic analysis. Indeed, peritumoral radiomics has shown promise in predicting non–small cell lung cancer subtypes (30), epithelial ovarian cancer extra-pelvic metastasis (31), and malignant brain tumor classification (32). However, its application to parotid gland tumors remains underexplored.

In this study, the diagnostic efficacy of intratumoral and peritumoral radiomic features for discriminating benign and malignant parotid gland tumors was systematically evaluated, revealing that the peritumoral microenvironment may harbor critical biological cues for tumor characterization. Likewise, the selection of peritumoral margin size significantly influences radiomic predictive performance (33), and the systematic identification of an optimal expansion zone can further enhance model accuracy. Although optimal peritumoral ranges vary by anatomical site, definitive evidence for parotid tumors has been lacking. By constructing models at 1 mm, 2 mm, 3 mm, and 4 mm peritumoral expansions, we sought to determine the ideal margin for parotid neoplasms and confirmed that peritumoral heterogeneity provides substantial complementary value in malignancy prediction. We integrated multiphase CT features—unenhanced, arterial, and venous—into nine distinct radiomics models and found the 2 mm expansion to be optimal, yielding AUCs of 0.927 and 0.791 in the training and test cohorts, respectively, outperforming the 1 mm, 3 mm, and 4 mm models. Furthermore, the combined intratumoral + 2 mm peritumoral model demonstrated superior discrimination (AUC = 0.956 in training, 0.836 in testing), with high accuracy and specificity. Consistent with Shen et al.’s findings that a 2 mm peritumoral margin maximizes AUC when compared to 5 mm (34), our results corroborate the primacy of the 2 mm zone. Prior research indicates that support vector machine (SVM)–based classifiers outperform other machine learning algorithms (18) and, owing to their robust generalizability, suitability for small datasets, and interpretability, we employed SVM throughout. Moreover, the Peri1 mm model also demonstrated robust diagnostic performance; likewise, the Peri3 mm (AUC = 0.983) and Peri4 mm (AUC = 0.976) models achieved high AUCs in the training cohort, yet their discriminative accuracy declined markedly in the test cohort. Moreover, we observed that standalone peritumoral models underperformed relative to intratumoral models, potentially reflecting our moderately sized cohort and the predominance of larger tumors, which inherently contain abundant discriminative information, thereby limiting the incremental value of peritumoral features.

The Intra + Peri2 mm model demonstrated a pivotal role in enhancing the predictive performance for parotid gland tumor characterization. By integrating intratumoral and 2 mm peritumoral radiomic signatures, the IntraPeri2 mm model achieved AUCs of 0.956 and 0.836 in the training and test cohorts, respectively. Consequently, we infer that intratumoral and peritumoral features are complementary, jointly conferring superior accuracy in distinguishing benign from malignant parotid lesions. Decision curve analysis further confirmed that the IntraPeri2 mm model yields a meaningful net clinical benefit.

Furthermore, this study identified clinical symptoms and tumor margin status as independent predictors of malignancy, and combined these clinical‐imaging variables with the optimal radiomic signature to construct a joint model. The resulting combined model attained AUCs of 0.974 in the training set and 0.844 in the test set—outperforming both the clinical‐only and radiomics‐only models—and was visualized via a nomogram. The integration of these clinical and radiomic parameters enables more accurate preoperative stratification of parotid gland tumors, thereby holding significant implications for individualized clinical decision-making.

## 5 Study limitations

This investigation is subject to several constraints. First, its retrospective design inherently predisposes it to selection bias. Second, as a single-center study with a modest and imbalanced sample, external validation was not feasible; the limited cohort may constrain the robustness and generalizability of our findings, underscoring the need for prospective, multicenter trials with larger, more heterogeneous populations. Third, manual delineation of tumor ROIs introduces intra- and interobserver variability; the implementation and validation of automated or semi-automated segmentation algorithms are warranted to enhance reproducibility and mitigate observer-dependent discrepancies.

## 6 Conclusion

Multiphase CT-derived radiomic signatures encompassing both intratumoral and peritumoral regions demonstrate high discriminatory power for benign versus malignant parotid gland tumors. Integration of these quantitative imaging biomarkers with independent clinical predictors yields a composite model with superior diagnostic accuracy and clinical net benefit. This noninvasive framework offers a promising avenue for precision stratification and individualized management of parotid neoplasms.

## Data Availability

The original contributions presented in the study are included in the article/supplementary material. Further inquiries can be directed to the corresponding author.
